# Treating asthma in patients with enuresis: repercussions on urinary symptoms

**DOI:** 10.1590/S1677-5538.IBJU.2023.0101

**Published:** 2023-07-20

**Authors:** Patricia Dahan, Pricila Mara Novais de Oliveira, Aparecida Regina Brum, André Costa Pinto Ribeiro, André Avarese Figueiredo, José de Bessa, José Murillo B.

**Affiliations:** 1 Faculdade de Medicina da Faculdade de Ciências Médicas e da Saúde de Juiz de Fora Departamento de Pediatria Juiz de Fora MG Brasil Departamento de Pediatria, Faculdade de Medicina da Faculdade de Ciências Médicas e da Saúde de Juiz de Fora (SUPREMA), Juiz de Fora, MG, Brasil; 2 Universidade Federal de Juiz de Fora Hospital Universitário Departamento de Fisioterapia Pediátrica Juiz de Fora MG Brasil Departamento de Fisioterapia Pediátrica do Hospital Universitário da Universidade Federal de Juiz de Fora (UFJF), Juiz de Fora, MG, Brasil; 3 Hospital Evandro Ribeiro Juiz de Fora MG Brasil Serviço de Otorrinolaringologia do Hospital Evandro Ribeiro, Juiz de Fora, MG, Brasil; 4 Universidade Federal de Juiz de Fora Departamento de Cirurgia do Hospital Universitário Departamento de Otorrinolaringologia Juiz de Fora MG Brasil Departamento de Otorrinolaringologia, Departamento de Cirurgia do Hospital Universitário da Universidade Federal de Juiz de Fora (UFJF), Juiz de Fora, MG, Brasil; 5 Universidade Federal de Juiz de Fora Departamento de Cirurgia Divisão de Urologia Juiz de Fora MG Brasil Departamento de Cirurgia- Divisão de Urologia, Universidade Federal de Juiz de Fora (UFJF), Juiz de Fora, MG, Brasil; 6 Universidade Estadual de Feira de Santana Departamento de Cirurgia Divisão de Urologia Feira de Santana Bahia Brasil Departamento de Cirurgia - Divisão de Urologia, Universidade Estadual de Feira de Santana, Feira de Santana, Bahia, Brasil; 7 Faculdade de Ciências Médicas e da Saúde de Juiz de Fora Departamento de Cirurgia Juiz de Fora MG Brasil Departamento de Cirurgia, Faculdade de Ciências Médicas e da Saúde de Juiz de Fora (SUPREMA), Juiz de Fora, MG, Brasil

**Keywords:** Enuresis, Asthma, Apnea

## Abstract

**Background:**

Children presenting enuresis are more likely to be asthmatics. The association between enuresis and sleep-disordered breathing has already been demonstrated and several studies have shown at least partial improvement of two thirds or more of the cases of enuresis adenoidectomy. Studies have already described associations between enuresis and allergies but do not assess the repercussions of allergy treatment in enuretics.

**Objective:**

This study aims to evaluated whether asthma treatment alters the course of enuresis and whether there is any predictive factor associated with this improvement.

**Materials and Methods:**

Twenty patients (5 - 12 years old) with uncontrolled enuresis and asthma, received treatment for asthma. Children were also assessed for the presence of rhinitis and other allergies. The control of asthma was confirmed by a validated questionnaire and primary enuresis by clinical history and wet night diaries. Patients received only asthma treatment.

**Results:**

At least partial improvement of enuresis was observed in 55% of the patients with an increase in 64.4% in the number of dry nights at the end of the study (p=0.01). The “presence of other allergies” and “obstruction seen in nasal endoscopy” positively influenced the improvement of urinary symptoms (OR = 3.350; CI 0.844–13.306) and (OR=1.272; CI 0.480-3.370), respectively.

**Discussion:**

Until now, only patients presenting upper airway obstruction were known to benefit from the improvement of urinary symptoms when undergoing treatment for their respiratory problems. In our study, we found at least partial improvement in enuresis in 55% of our patients, with only clinical asthma treatment.

**Conclusion:**

Controlling asthma in children with primary enuresis resulted in a significant increase in dry nights.

## INTRODUCTION

Enuresis is characterized by the involuntary loss of urine during sleep in children five years of age and older. According to the criteria of the International Children’s Continence Society (ICCS), the definition of enuresis is established when there is at least one episode of urinary loss per month for at least three consecutive months, and after ruling out other organic diagnoses (1). The prevalence of enuresis is approximately 5 to 10% in children with seven years of age and 3% in adolescents with a great impact in quality of life (2).

Asthma is a heterogeneous disease caused by chronic inflammation of the lower airways (LOA) and clinically manifests with recurrent episodes of wheezing, dyspnea, tightness of the chest and coughing. The WHO lists asthma prevalence rates from 1 to 18% in different countries (3). Sleep-disordered breathing (SDB) are associated with upper airway obstruction (UAO). At the extremes are primary snoring, consisting only of respiratory noise, and Obstructive Sleep Apnea Syndrome (OSAS), in which there are repeated episodes of UAO collapse with drops in oxygen saturation (4). Epidemiological studies indicate a prevalence of 10 to 27% of primary snoring and 0.5 to 9% of apneas reported by parents (5).

The association between enuresis and SDB has already been demonstrated in several studies (6–9). Various studies have shown resolution of two thirds or more of the cases of enuresis following surgical resolution of SDB through an adenoidectomy, with or without tonsillectomy (6–9). Several pathophysiological mechanisms have been proposed to explain the association between enuresis and SDB. The main one is the release of brain natriuretic peptide (BNP) by cardiac cells after atrial wall distention due to increased negative intrathoracic pressure in upper respiratory obstruction. The increase in BNP levels is responsible for the increase in sodium and water excretion, in addition to inhibiting the release of vasopressin and the renin-angiotensin-aldosterone system (10). In addition, partial or complete UAO events in SDB, micro-recurrent awakenings caused by obstruction of the upper airways lead to an increased threshold of awakening and stimulation such as bladder filling or contractions of the detrusor muscle become ineffective. (9, 10).

The association between enuresis and asthma at some point in life was described by Ra-washdeh et al. (2002) (11). This association was also demonstrated by our group in schoolchildren from 6 to 14 years of age. We showed that children with enuresis are 2.8 times more likely to be asthmatic (8). Recently, a study carried out in Turkey confirmed a higher prevalence of enuresis in asthmatic children (12).

It is questioned whether this association found between enuresis and asthma is related to the concomitant presence of anatomic obstruction of the upper airway, as in OSAS. Abnormalities of the UAO and LOA can coexist according to the united airway hypothesis (13). Epidemiological studies have already confirmed the association between allergic rhinitis and asthma (14). Likewise, wheezing children have a higher incidence of snoring and nocturnal apnea. Apnea severity is associated with asthma severity, and the apnea- hypopnea index (AHI) is higher in children with uncontrolled asthma (15).

Since the association between asthma and enuresis is related to the presence of concomitant OSA, the resolution of UAO in enuretic and asthmatic patients would be the only determining factor for the resolution of urinary and asthma symptoms. This hypothesis, however, does not clarify the association between enuresis and a history of allergy confirmed by skin tests found by Rawash- deh et al. (2002) (11).

Several studies associate enuresis with allergic conditions such as urticaria, seasonal rhinitis, or drug or food allergy (11, 16, 17). The risk of enuresis is 1.7 times greater in patients with allergic rhinitis, and this risk was higher in boys and patients with comorbidities such as asthma and topic dermatitis. (11, 18). These studies do not assess the repercussions of allergy treatment in enuretics patients with respiratory symptoms. Assuming that the treatment of respiratory allergies can influence the improvement of enuresis, we would have the possibility of shortening the approach in this group of patients with both diseases.

Therefore, this study aims to assess whether asthma treatment alters the course of primary enuresis in children from 5 to 12 years of age with both diseases, and whether “upper airway obstruction”, the “presence of rhinitis or other allergies reported by parents” and “obesity” are predictive of this improvement in urinary symptoms with asthma treatment.

## MATERIAL AND METHODS

A prospective, uncontrolled interventional study was conducted, in which 20 patients from 5 to 12 years of age with uncontrolled asthma and with primary enuresis received inhaled treatment for asthma between September 2019 and April 2022.

This study was approved by the Human Research Ethics Committee (66961717.7./0000.51.03) and all parents and children signed a free an informed consent and ascent form, respectively.

The recruitment of participants with uncontrolled asthma and concurrent enuresis was carried out in pediatric pulmonology and pediatric urology outpatient clinics, in addition to an active search for patients at the level of public schools and basic health units, through posters placed with telephone contact. Only patients over 5 years and under 15 years of age, with current uncontrolled asthma and current enuresis were eligible to participate.

In order to define the presence of enuresis we followed the criteria of ICCS i.e., the answer “yes” by the parent to the question: “has your child wet the bed at night at least once a month for at least three consecutive months?”. Only children with primary enuresis (PE) participated in the project (1).

To define current asthma, parents initially answered the following question from the International Study of Asthma and Allergies in Childhood (ISAAC) questionnaire (19): “In the last 12 months, has your child had wheezing in the chest?” (20). Those who answered “yes” were then asked about the level of disease control in the last month. To select children with uncontrolled asthma, we used the Global Initiative for Asthma criteria (3).

The exclusion criteria were: age under five years, presence of diseases and/or use of medications that may interfere with the functioning of the bladder or urethral sphincter, diabetes, facial malformations and/or genetic syndromes with upper airway obstruction, secondary enuresis, controlled asthma, or refusal to participate in the study.

At the first consultation with the researcher, the selected patient and his/her guardian answered a detailed medical history for asthma prepared by the author herself, as well as for enuresis. The clinical diagnosis of allergic rhinitis was defined through the presence of symptoms such as nasal itching, nasal congestion, runny nose, sneezing, itchy eyes, or others, such as throat clearing. Other allergies such as urticaria without a defined cause, food or drug allergy, strophulus, anaphylactic shock, sudden swelling of the eyes and/or mouth and atopic dermatitis lesions were also questioned. The assessment of the level of asthma control in the last month was carried out in children from 5 to 11 years of age using the Childhood Asthma Control Test (c-ACT) questionnaire and the Asthma Control Test (ACT) questionnaire for children over 12 years of age.

The patients were then consulted at a pediatric urology outpatient clinic, where their guardians were instructed on the collection of the three- day voiding diary and the Dry Nights Diary, which corresponds to a record of dry or wet nights for 14 days. In the voiding diary, the child and their guardians recorded all episodes related to fluid intake and urination, as well as urgency and incontinence, for three days.

All patients underwent nasal endoscopy performed by the same otolaryngologist to diagnose and measure a possible cause of upper respiratory obstruction, such as adenoid hypertrophy. The examination was performed with a Pentax® 10RP3 fiber naso-pharyngo-laryngoscope and the classification of UAO described in percentage of UAO: from 0-25%; 25-50%; 50-75% and equal to or greater than 75% according to the Brodsky classification used to assess palatine tonsils (21).

Asthma treatment was started after the first interview, nasal endoscopy and collection of diaries. For that, inhaled beclomethasone was used at a dose of 200 mcg every 12 hours, administered through a spacer with mouthpiece, a device that allows for the application of the aerosol and minimizes problems of inadequate inhalation (22).

Subsequent consultations were monthly and, in addition to a physical examination (determination of BMI), it was included an assessment of the inhalation technique to avoid possible medication application errors, the patient’s level of asthma control and the frequency of enuresis assessed by collecting a diary of continuous dry and wet nights. If the level of control indicated uncontrolled asthma (score less than 20 points on the c-ACT or ACT questionnaires) in any of the subsequent visits, even with continuous treatment and the inhalation mechanism performed properly, then the treatment was adjusted to a high dose of inhaled corticosteroid associated with a long-acting bronchodilator (salmeterol and fluticasone at a dosage of 25/125 mcg, two sprays every 12 hours) (21).

**Figure 1:**
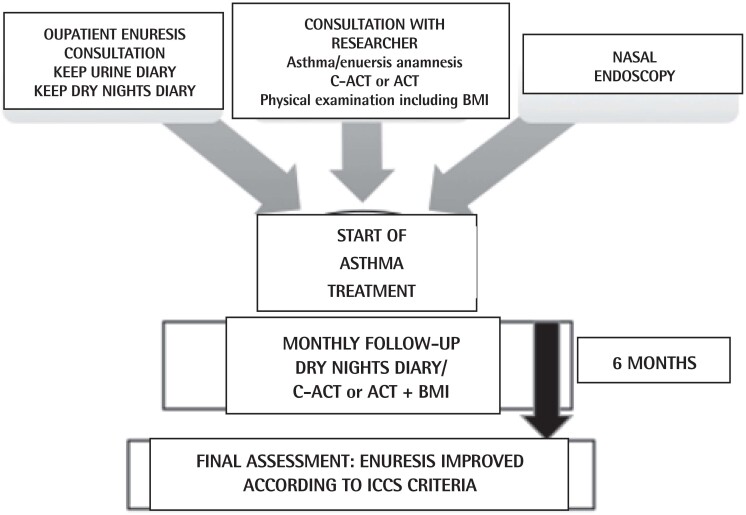
Description of the project stages from admission to the end of the survey.

The project stages are listed in Figure-1.

Through a diary of dry and wet nights, we defined the number of dry nights over 30 days at the entrance and exit of the survey. We consider any improvement (partial and total improvement) above 50% improvement as per ICCS criteria (1).

Quantitative, continuous, or ordinal variables were described by their measures of central tendency (means or medians) while nominal or qualitative variables were described by their absolute values, percentages, or proportions. We used the Student’s t test to compare the difference of the main continuous variable (number of dry/wet nights). In order to assess the measures of the effects of the independent variables (UAO; presence of allergies and obesity) with the binomial variable (improvement of enuresis according to ICCS criteria) we used logistic regression analysis. The statistical program Jamovi (Version 1.6) was used for the analyses. The strategy for selecting the variables was forward and the interpretation of the models was based on the analysis of the odds, AIC, deviance and R2 squared.

## RESULTS

Thirty-two children with current primary enuresis and uncontrolled asthma who met the inclusion criteria for the study were recruited; of these, 12 dropped out of the study in the first months of treatment or even before starting it.

Therefore, a total of 20 children, 5 to 12 (7.4 ± 2.41) years of age, being 10 boys completed the entire six-month follow-up period. Five patients (25%), all boys, were obese for their age. All patients had symptoms of allergic rhinitis, as well as at least some other type of allergy (Table-1). The results of the nasal endoscopy described that 70% of the children had at least an UAO of 50% with 60% upper than 75%. A total of 15 children had non- monosymptomatic enuresis and the symptoms related were: urgency, urge incontinence, increased or infrequent micturition.

**Table 1:** Characteristics of the population studied.

Characteristics assessed	n (%)
Obesity	5 (25%)
Male	5 (100%)
Female	0 (0%)
Presence of allergic rhinitis symptoms	20 (100%)
Non-monosymptomatic enuresis	15 (75%)
Report of other allergies (food, drug, etc.)	20 (100%)
One allergy	6 (30%)
Two allergies	7 (35%)
Three allergies	5 (25%)
Four or more allergies	2 (10%)

All patients presented control of their asthma symptoms in the first 2 months of treatment and 7 of the 20 patients (35%) required the addition of long-term bronchodilator (salmeterol) to the inhalation corticosteroid to achieve this control. There was no correlation between the need for bronchodilator addition with improvement in urinary symptoms. After six months of asthma treatment and according to the ICCS classification, three patients had complete improvement of enuresis (improvement of urinary symptoms greater than 99%) and eight patients had partial improvement of enuresis (50 to 99% resolution of symptoms). Therefore, 55% of the patients had at least a partial response (50% or more reduction) in enuresis during the course of the study.

**Figure 2:**
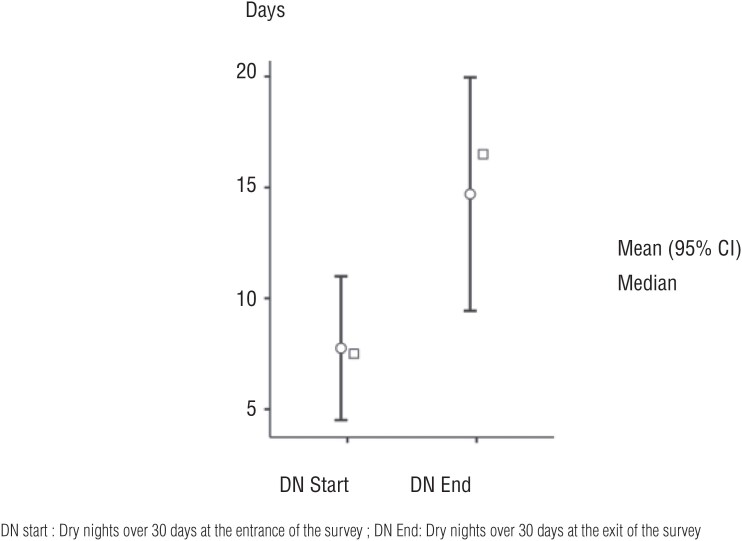
Increase of the number of dry nights over 30 days at the entrance and exit of the survey.

A mean increase in dry nights of 64.4% (p<0.01) over 30 days was observed at the end of treatment (Figure-2).

Through logistic regression, the presence of other allergies and obstruction in nasal endoscopy positively influenced this improvement (OR=3.350; CI 0.844-13.306) and (OR=1.272; CI 0.480-3.370). The independent variable “obesity” did not influence the improvement in enuresis of the patients (OR = 0.361; CI 0.023–5.641) (Table-2)..

**Table 2:** More stable binomial logistic regression model.

Binomial logistic regression		Deviance 21.4		AIC 31.4	R2 0.224	
Predictor	Estimate	95% Confidence Interval				
		Lower	Upper	SE	p	OR
Obesity	1.0201	-3.770	1.7301	1.4032	0.467	0.361
Allergies	1.2089	-0.170	2.5882	0.7037	0.086	3.350
Nasal endoscopy	0.2405	-0.734	1.2151	0.4973	0.629	1.272
WN in 30 days	0.1165	-0.279	0.0457	0.0828	0.159	0.089

Estimates represent the log odds of “CLASSIF. ICCS = 1” (improvement of enuresis) vs. “CLASSIF. ICCS = 0” (no improvement) AIC=AKAIKE criteria; OR=Odds ratio; SE (Standard error); WN = wet nights

## DISCUSSION

The results presented here show an improvement in enuresis with the treatment of asthma in patients with both diseases and, therefore, bring a new treatment perspective for this group of children presenting both enuresis and respiratory symptoms. Until now, only patients presenting upper airway obstruction were known to benefit from the improvement of urinary symptoms when undergoing treatment for their respiratory problems, as described that surgical intervention for UAO can treat up to two thirds of children presenting enuresis (6–9).

In our study, we found at least partial improvement in enuresis in 55% of our patients without any surgical intervention, with only clinical asthma treatment. The decrease in wet nights over 30 days, at the beginning and end of the study, was 35.6%.

Nocturnal enuresis has a spontaneous resolution rate of 15% per year. We followed our patients for about half a year and we were unable to differentiate spontaneous improvement from improvement with the proposed treatment. However, our results seem to be superior to spontaneous resolution (15% total resolution and 40% partial resolution in 6 months).

We know that not all children with enuresis and SDB who undergo surgical correction of upper airway obstruction show improvement in urinary symptoms. Some authors have tried to elucidate the reason why some respond and others do not. It was observed that the response is better in those whose preoperative sleep study shows greater severity of apnea (greater number of obstructive events and awakenings) (23). Due to the difficult access to the sleep study exam, we defined the degree of obstruction of the upper airways of our patients through flexible endoscopy and, therefore, we do not have information on the clinical repercussion of this obstruction during sleep (apnea and hypopnea index). The endoscopic evaluation is momentary and we assume that the obstruction found may be overestimated by the edema found in situations of allergic rhinitis.

Among the factors that positively influenced the improvement of enuresis according to ICCS criteria, we had the presence of allergies other than rhinitis and asthma. All 20 patients reported symptoms of allergic rhinitis, which made it impossible to use this variable.

The association of enuresis with allergies is already well demonstrated in the literature (11, 12, 16–19). In a case-control study conducted in Turkey, 34% of enuretic patients presented with allergic illnesses, while these occurred in only 12% of the control group (24). Likewise, the association of enuresis with urticaria and food or drug allergies has been described in enuretic boys (16). These studies are observational and only show an association between atopy (allergic rhinitis, food allergies, urticaria, among others) with the presence of enuresis. There are no current reports of interventional studies to assess the repercussion of the treatment of allergic conditions on the symptoms of enuresis.

Likewise, we observed a positive association of the factor “obstruction seen in nasal endoscopy” with the improvement of enuresis after asthma treatment. This result initially surprised us since most studies that show an association between enuresis and obstructive sleep apnea in children also show improvement in urinary symptoms in most patients after a surgical procedure (adenoidectomy and/or tonsillectomy) (6–9). What may explain this positive association between the “obstruction seen in nasal endoscopy” and improvement in enuresis with asthma treatment would be the concomitant presence of allergic rhinitis (AR). In the literature, 60.8% of patients with adenoid hypertrophy have associated allergic rhinitis (25).

AR is associated with a higher risk of sleep disorders, such as insomnia or restless sleep, enuresis and SDB (both OSA and primary snoring) (25). It is now believed that rhinitis potentiates SDB and is even an independent predictor of unsuccessful adenotonsillectomy in children (26). In our study, 60% of the patients had an obstruction greater than 75% on nasal endoscopy and all patients reported symptoms of allergic rhinitis. The concomitant presence of AR was probably an aggravating factor in the results of the endoscopic examination.

As with asthma and enuresis, allergic rhinitis also compromises sleep (27). Some inflammatory mediators involved in this allergic process have been associated with sleep abnormalities verified in sleep studies: increased latency and decreased Rapid Eye Movement sleep time, as well as decreased latency for the onset of sleep (27). The sleep of enuretics is more fragmented, which leads to consequent sleep deprivation and collaborates to increase the awakening threshold of sleep (28). Likewise, sleep quality in asthma is inversely proportional to disease control. Worse objective measures of lung function, such as FEV1 (Forced Expiratory Volume in the first second), defined by spirometry, are associated with worse sleep quality (29). Therefore, asthma, AR, SDB and enuresis compromise patients’ sleep in some way. It remains to be seen whether the control of any of these sleep disorders can retroactively interfere with the others, only through improved sleep quality.

A retrospective study in 393 children with primary enuresis monosymptomatic describe that winter season was associated with desmopressin treatment failure. We did not observe possible interference from colder weather due to the Covid-19 pandemic emergence and its protective measures during much time of the study (30).

Enuresis as a possible adverse effect of using topical corticosteroids for allergic rhinitis or long-acting bronchodilators for asthma has been described for the use of intranasal corticosteroids; enuresis was observed in a minority of children (3 cases in 106 children) and it does not correspond to the treatment route used in our study (31). Only 7 of the 20 patients required the combination of salmeterol with fluticasone to control their asthma. There was no correlation between the need for bronchodilator addition with improvement in urinary symptoms. All patients were instructed to use a spacer for asthma treatment and to perform posterior oral hygiene. These mechanisms decrease the systemic absorption of the medication.

One of the limitations found in the study was the high dropout rate (37.5%). We associate this occurrence with a behavior that is common to many patients with chronic diseases who require constant care (3). Another limitation was the impossibility of adding a control group: we understand that making a diagnosis of uncontrolled asthma and not providing the recommended treatment for it would be unethical.

Finally, it is clear that many studies are still needed to clarify the effectiveness of the clinical treatment of allergies on the repercussions of enuresis symptoms. However, we suggest that, in the future, a flowchart for the initial approach of enuretic patients with respiratory symptoms should be proposed. We could thereby direct the treatment of these patients, preventing them from being subjected to long and unsatisfactory treatments, generating frequent dropouts.

## CONCLUSIONS

Treating and controlling asthma in children with primary enuresis resulted in a significant increase in dry nights. The presence of upper airway obstruction and other allergies positively improved results.

## References

[1] Nevéus T, Fonseca E, Franco I, Kawauchi A, Kovacevic L, Nieuwhof-Leppink A (2020). Management and treatment of nocturnal enuresis-an updated standardization document from the International Children’s Continence Society. J Pediatr Urol.

[2] Rangel RA, Seabra CR, Ferrarez CEPF, Soares JL, Choi M, Cotta RG (2021). Quality of life in enuretic children. Int Braz J Urol.

[3] [No Authors], Who Health Organization (2020). Global Strategy for Asthma Management and Prevention. Global Initiative for Asthma.

[4] Witmans M, Young R (2011). Update on pediatric sleep-disordered breathing. Pediatr Clin North Am.

[5] Kaditis AG, Finder J, Alexopoulos EI, Starantzis K, Tanou K, Gampeta S (2004). Sleep-disordered breathing in 3,680 Greek children. Pediatr Pulmonol.

[6] Aydil U, Işeri E, Kizil Y, Bodur S, Ceylan A, Uslu S (2008). Obstructive upper airway problems and primary enuresis nocturna relationship in pediatric patients: reciprocal study. J Otolaryngol Head Neck Surg.

[7] Weissbach A, Leiberman A, Tarasiuk A, Goldbart A, Tal A (2006). Adenotonsilectomy improves enuresis in children with obstructive sleep apnea syndrome. Int J Pediatr Otorhinolaryngol.

[8] Dahan P, de Bessa J, de Oliveira DM, Gomes CC, Cardoso JC, Macedo IT (2016). Association between Asthma and Primary Nocturnal Enuresis in Children. J Urol.

[9] Ribeiro A, Bastos JM, de Figueiredo AA, Cândido TC, Guércio WB, Zica BO (2022). Enuresis and upper airway obstruction: BNP and ADH hormones behavior before and after airway surgery. Int Braz J Urol.

[10] Sans Capdevila O, Crabtree VM, Kheirandish-Gozal L, Gozal D (2008). Increased morning brain natriuretic peptide levels in children with nocturnal enuresis and sleep- disordered breathing: a community-based study. Pediatrics.

[11] Rawashdeh YF, Hvistendahl GM, Kamperis K, Hansen MN, Djurhuus JC (2002). Demographics of enuresis patients attending a referral centre. Scand J Urol Nephrol.

[12] Ozkaya E, Aydın SC, Yazıcı M, Dundaröz R (2016). Enuresis Nocturna in children with asthma: prevalence and associated risk factors. Ital J Pediatr.

[13] Braunstahl GJ (2009). United airways concept: what does it teach us about systemic inflammation in airways disease?. Proc Am Thorac Soc.

[14] Ramagopal M, Mehta A, Roberts DW, Wolf JS, Taylor RJ, Mudd KE (2009). Asthma as a predictor of obstructive sleep apnea in urban African-American children. J Asthma.

[15] Chan CS, Woolcock AJ, Sullivan CE (1988). Nocturnal asthma: role of snoring and obstructive sleep apnea. Am Rev Respir Dis.

[16] Zaleski A, Shokeir MK, Gerrard JW (1972). Enuresis: familial incidence and relationship to allergic disorders. Can Med Assoc J.

[17] Mungan NA, Seckiner I, Yesilli C, Akduman B, Tekin IO (2005). Nocturnal enuresis and allergy. Scand J Urol Nephrol.

[18] Lai PH, Yang PS, Lai WY, Lin CL, Hsu CY, Wei CC (2018). Allergic rhinitis and the associated risk of nocturnal enuresis in children: a population-based cohort study. Int Forum Allergy Rhinol.

[19] Asher MI, Keil U, Anderson HR, Beasley R, Crane J, Martinez F (1995). International Study of Asthma and Allergies in Childhood (ISAAC): rationale and methods. Eur Respir J.

[20] Solé D, Vanna AT, Yamada E, Rizzo MC, Naspitz CK (1998). International Study of Asthma and Allergies in Childhood (ISAAC) written questionnaire: validation of the asthma component among Brazilian children. J Investig Allergol Clin Immunol.

[21] Brodsky L (1989). Modern assessment of tonsils and adenoids. Pediatr Clin North Am.

[22] [No authors] (2012). Diretrizes da Sociedade Brasileira de Pneumologia e Tisiologia para o manejo da asma. J Bras de Pneumol.

[23] Kovacevic L, Wolfe-Christensen C, Lu H, Toton M, Mirkovic J, Thottam PJ (2014). Why does adenotonsillectomy not correct enuresis in all children with sleep disordered breathing?. J Urol.

[24] Yılmaz-Durmuş S, Alaygut D, Soylu A, Alparslan C, Köse SŞ, Anal Ö (2018). The association between monosymptomatic enuresis and allergic diseases in children. Turk J Pediatr.

[25] Liu J, Zhang X, Zhao Y, Wang Y (2020). The association between allergic rhinitis and sleep: A systematic review and meta-analysis of observational studies. PLoS One.

[26] Huo Z, Shi J, Shu Y, Xiang M, Lu J, Wu H (2017). The relationship between allergic status and adenotonsillar regrowth: a retrospective research on children after adenotonsillectomy. Sci Rep.

[27] Krouse HJ, Davis JE, Krouse JH (2002). Immune mediators in allergic rhinitis and sleep. Otolaryngol Head Neck Surg.

[28] Cohen-Zrubavel V, Kushnir B, Kushnir J, Sadeh A (2011). Sleep and sleepiness in children with nocturnal enuresis. Sleep.

[29] Koinis-Mitchell D, Kopel SJ, Seifer R, LeBourgeois M, McQuaid EL, Esteban CA (2017). Asthma-related lung function, sleep quality, and sleep duration in urban children. Sleep Health.

[30] Sun M, Li S, Sun X, Deng Z, Xu Y (2022). Association between winter season and desmopressin treatment efficiency in children with monosymptomatic nocturnal enuresis: a pilot study. Int Braz J Urol.

[31] Rollema C, van Roon EN, Ekhart C, van Hunsel FPAM, de Vries TW (2022). Adverse Drug Reactions of Intranasal Corticosteroids in the Netherlands: An Analysis from the Netherlands Pharmacovigilance Center. Drugs Real World Outcomes.

